# Evaluation of mast cells and hypoxia inducible factor-1 expression in meningiomas of various grades in correlation with peritumoral brain edema

**DOI:** 10.1007/s11060-013-1208-1

**Published:** 2013-07-23

**Authors:** Joanna Reszec, Adam Hermanowicz, Robert Rutkowski, Piotr Bernaczyk, Zenon Mariak, Lech Chyczewski

**Affiliations:** 1 Department of Medical Pathomorphology, Medical University of Bialystok, Waszyngtona 13, 15-269 Bialystok, Poland; 2 Department of Neurosurgery, Medical University of Bialystok, Skłodowskiej-Curie 24A, 15-276 Bialystok, Poland

**Keywords:** Meningioma, Mast cells, Hypoxia inducible factor-1

## Abstract

Meningiomas are common primary brain tumors. However, they are often complicated by significant peritumoral brain edema, which leads to surgery difficulties and prolonged hospitalization. The aim of this study was to evaluate the presence of mast cells and expression of hypoxia inducible factor-1 (HIF-1) in correlation with the grade of meningioma and presence of peritumoral brain edema. Immunohistochemistry was performed with specific antibodies against tryptase (mast cells) and HIF-1 in low grade meningiomas (estimated as G1) and high grade meningiomas (estimated as G2 or G3). Peritumoral brain edema observed in MRI was graded using Steinhoff classification. Tryptase expression was observed in 40.4 % low grade meningiomas and in 90 % high grade cases; HIF-1 in 55.7 % low grade and in 84 % high grade meningiomas. There was a statistically significant correlation between HIF-1 and tryptase expression in both groups (*p* = 0.003). Presence of peritumoral brain edema statistically correlated with tryptase (*p* = 0.001) and HIF-1 expression (*p* = 0.004). Mast cells as well as hypoxia are involved in meningioma progression, and may be associated with the formation of peritumoral brain edema leading to surgery complication and recovery. Therefore, they may be useful markers in predicting the clinical course of meningioma cases.

## Introduction

Mast cells are multi-effector cells, however they are still the least understood components of the immune system [1]. In recent decades, the importance and fascinating biology of mast cells has been described in many physiological and pathological conditions, but the role of mast cells in tumor development and progression is still unknown [2]. Mast cells (MCs) are often present in large amounts in the stroma of different neoplasms, such as: mammary adenocarcinoma, colorectal adenocarcinoma, urothelial carcinoma, neurofibroma or melanoma [3]. Much data postulated that MCs may affect tumor development in few different ways: by affecting tumor cells directly, by modulating the tumor microenvironment, or by activating other inflammatory cells beneficial for tumor progression [3, 4]. The effect of that activity depends on the multiplicity of molecules which are synthesized and secreted to the extracellular matrix from MCs’ granules [5], such as: platelet derived factor, colony stimuli factor, and nerve growth factor [6]. Also histamine could affect tumor progression by inducing proliferation through H1 receptor and suppressing the immune system via the H2 receptor. It was discovered that histamine and tryptase concentrations positively correlate with mast cell count in mammary carcinomas [7].

It is also known that tumor growth and progression is closely related to angiogenesis [5, 6]. Pathological angiogenesis mainly depends on the release of specific growth factors for endothelial cells, which may stimulate proliferation of the host’s blood vessels. Some data suggest that activated MCs synthesize and secrete pro-angiogenic factors such as fibroblast growth factor-2 and vascular endothelial growth factor (VEGF). There is high expression of VEGF in MC accumulated around tumors [2, 4, 6].

There are many theories about brain tumor progression and the presence of mast cells. It depends on the blood–brain-barrier (BBB) [8, 9]. On the one hand, mast cells surround endothelial cells and, under the normal conditions, pericytes play the role of “gate keepers” of the BBB [10]. MCs also have anatomical and functional connections with neurons. On the other hand, acute stress can activate MCs by corticotrophin-releasing hormone and leads to inflammation development and increased BBB permeability, which may be responsible for brain metastases [7, 10].

Peritumoral brain edema in primary brain tumors and in metastases is a very serious complication associated with surgery and postsurgical recovery. Pathological vessels inside the tumor, without normal tight junctions, also lead to an increase of peritumoral edema, which is often observed in glioblastoma cases [8, 11, 12]. As a result, the surrounding edematous brain tissue might be easily infiltrated by neoplastic cells, resulting in tumor spreading.

Hypoxia-inducible factor-1 (HIF-1) is a transcriptional factor which consists of two subunits; under hypoxic conditions it may induce transcription of various genes involved in tumor angiogenesis (like vascular endothelial growth factor- VEGF), invasion (metalloproteinases), cell survival (antiapoptotic proteins), and glucose metabolism insulin growth factor receptor activation [12]. Overexpression of HIF-1 has been demonstrated in many common human cancers and correlated with tumor grade and progression, including renal, colon, and glioblastoma [12]. However, the significance of HIF-1 expression especially in high grade meningiomas is still unclear.

Almost 60 % of meningioma cases have peritumoral brain edema, mostly vasogenic edema with disruption of the BBB [8, 9, 13, 14]. Some of researchers suggest that peritumoral brain edema, resulting in worse prognosis or difficulties with total resection, is associated with hypoxia and inflammatory cell infiltration [13–15].

Therefore, the aim of this study was to evaluate the presence of mast cells, HIF-1 expression in association with peritumoral brain edema in meningiomas of various grades.

## Materials and methods

Peritumoral brain edema was evaluated in MRI by an experienced radiologist and graded by Steinhoff classification: 0—no signs of the edema; I- peritumoral brain edema limited to 2 cm; II- peritumoral brain edema limited to half of the hemisphere; III- more than half of the hemisphere [16].

We studied 154 meningioma cases at the Department of Neurosurgery Medical University of Bialystok (2008–2012). All cases were diagnosed by a neuropathologist based on WHO classification (Lyon 2007). Evaluation of mast cells presence and HIF-1 expression was performed using immunohistochemical methods. Following deparaffinization and rehydration, epitope retrieval was carried out in Envision Flex Target Retrieval Solution (DAKO) in high pH. Endogenous peroxidases were blocked by incubating sections in methanol and 3 % hydrogen peroxydase for 20 min. Next, slides were incubated with a typical mast cell marker, tryptase. We used monoclonal mouse anti-human mast cell tryptase (clone AA1, DAKO) in 1:100 dilution at room temperature for 20 min. The complexes were visualized with EnVision (DAKO) and DAB for 10 min. HIF-1 expression was evaluated using goat monoclonal antibody against HIF-1 protein (Santa Cruz Biotechnology HIF-1α, sc-53546) in 1:100 dilution stored overnight in 4 °C. Visualization reagent EnVision (DAKO) was applied for 30 min followed by DAB solution for 10 min. The slides were then counterstained with hematoxylin and examined under a light microscope. In order to avoid false evaluation, immunohistochemical estimation of each protein expression was performed by two independent pathologists. The intensity of immunostaining was evaluated in ten random fields under 20× magnification. The results were expressed as the percentage of cells with strong positive staining as follows: ≤10 % positive cells- negative (−), between 11 and 50 % (+), and ≥51 % positive cells (++) [17].

Appropriate positive and negative controls were performed. Negative controls were performed using a nonimmunized IgG replacing the primary antibody. Knowing tryptase expression in skin mastocytosis, we used those specimens as a positive control; for HIF-1 expression, renal clear cell cancer specimens were used.

All the procedures were approved by the local bioethics committee and conducted in accordance with institutional guidelines in compliance with national and international laws and guidelines.

### Statistical analysis

Chi squared, Pearson’s correlation test, and Statistica 10.0 StatSoft software were used for statistical analysis. Values of *p* < 0.05 were considered statistically significant.

## Results

In our present study, 104 tumors were diagnosed as low grade meningioma (G1), 50 were estimated as high grade meningioma (G2 or G3). All cases were classified according to WHO classification (Lyon 2007).

**Fig. 1:**
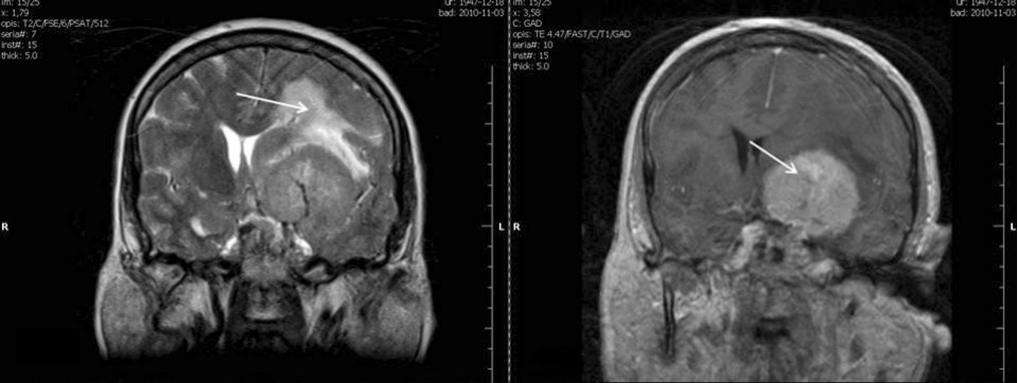
MRI scans showing peritumoral brain edema associated with meningioma, estimated (using Steinhoff classification) as grade II

Peritumoral brain edema was observed in 103 out of 154 meningiomas (66.8 %). 57 cases presented small peritumoral brain edema limited to 2 cm (I° Steinhoff classification), 44 covered less than half of the hemisphere (II° Steinhoff classification), and only two presented extensive peritumoral edema covering more than half of the hemisphere (III° Steinhoff classification) (Fig. 1). All patients with II° and III° had longer recovery time (from 4 to 10 days) compared with patients with I° or without peritumoral brain edema (from 3 to 7 days). Hospitalization time in the neurosurgery department was on average 2–3 days longer compared with patients classified as grade I meningiomas. After surgery, patients were admitted to the radiotherapy department for further treatment.

Low grade meningiomas were diagnosed in 27 male patients and in 77 female patients. Mean age in low grade meningiomas was 54.5 years.

High grade meningiomas were diagnosed in 27 male and in 23 female patients. Mean age was 52 years. The differences between age and sex in both groups were statistically significant (*p* = 0.003).

### Tryptase expression in both examined groups

**Fig. 2:**
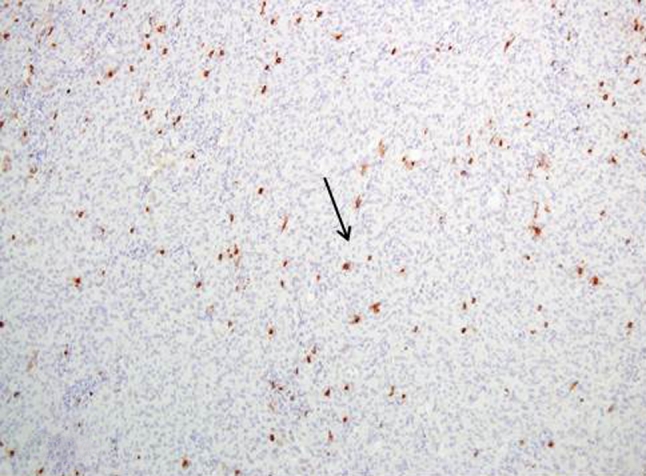
Tryptase expression in low grade meningioma Magn. ×100

**Fig. 3:**
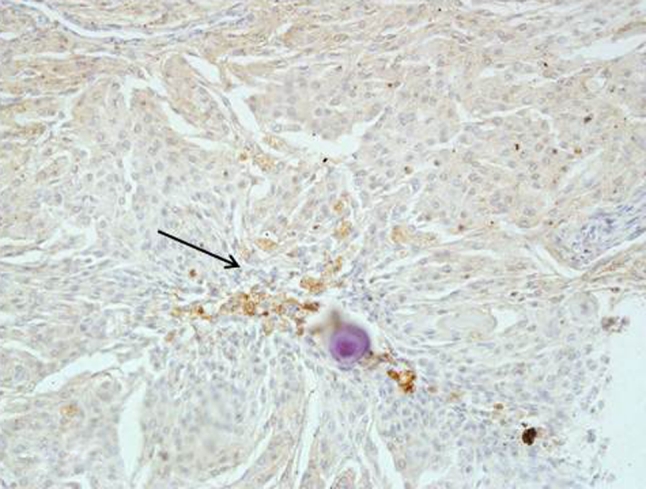
Low grade meningioma with patchy tryptase expression next to the psammoma body Magn. ×200

Tryptase expression was observed in 42 out of 104 low grade meningioma cases (40.4 %). 17 out of all positive specimens were estimated as (++) (Figs. 2, 3)—immunostaining was strong and observed in more than 50 % of neoplastic cells.

**Fig. 4:**
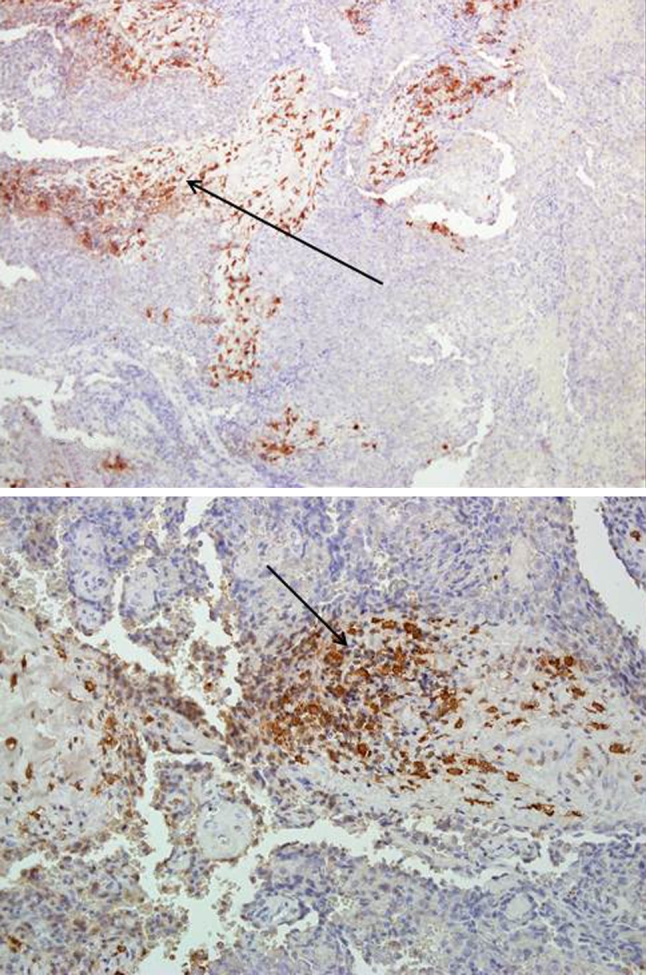
Intense tryptase expression in high grade meningioma observed mainly next to the blood vessels Magn. ×40, ×100

In high grade meningiomas, tryptase expression was observed in 45 out of 50 cases (90 %), 6 out of 45 were estimated as (++). Mast cells were observed not only next to blood vessels, but also within the tumor (Fig. 4).

### HIF-1 expression in both examined groups

HIF-1 expression was observed within the nucleus of neoplastic cells.

**Fig. 5:**
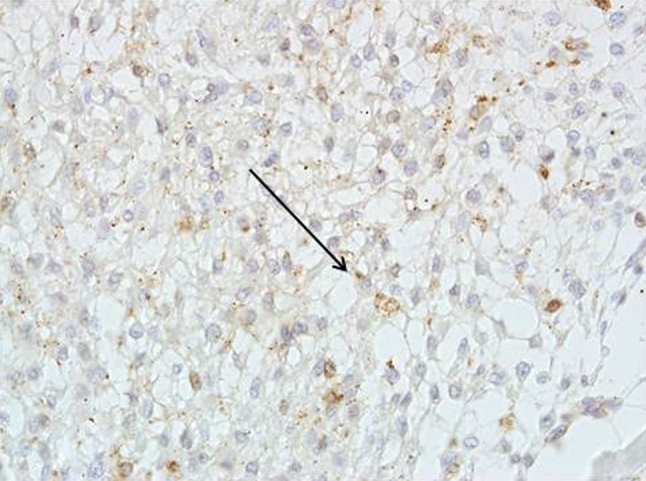
HIF-1 expression in low grade meningioma Magn. ×200

In low grade meningiomas, HIF-1 immunostaining was observed in 58 out of 104 cases (55.7 %) (Fig. 5).

**Fig. 6:**
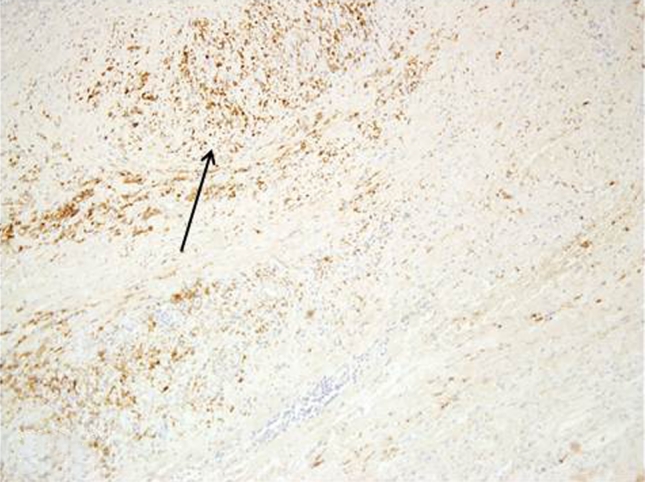
HIF-1 expression in high grade meningioma Magn. ×40

In the high grade meningioma group, HIF-1 expression was observed in 42 out of 50 cases (84 %) (Figs. 3, 6).

When we compared the expression of the examined marker with the degree of meningioma, we observed a statistically significant cross-correlation between tryptase expression and HIF-1 expression in both histological groups of meningiomas (Table 1) (*p* = 0.003).

**Table 1:** HIF-1 and tryptase coexpression in correlation with meningioma grade

Meningioma grade	Tryptase expression (−) (%)		Tryptase expression (+) (%)		Tryptase expression (++) (%)		*p* value
Low grade meningioma (G1) *n* = 104							*p* = 0.003
HIF-1 (−)	29	27.1	11	10.7	6	5.8	
HIF-1 (+)	32	31.1	14	13.6	10	9.6	
HIF-1 (++)	1	1.0	0	0.0	1	1.0	
High grade meningioma (G2 or G3) *n* = 50							
HIF-1 (−)	3	6.0	5	10.0	0	0.0	
HIF-1 (+)	2	4.0	34	68.0	5	10	
HIF-1 (++)	0	0.0	0	0.0	1	2.0	

In the low grade meningioma group, 25 out of 104 cases were positive for HIF-1 and tryptase; in the high grade meningioma group, 40 out of 50 cases presented positive staining for HIF-1 and tryptase.

### Correlation between tryptase, HIF-1 expression and presence of peritumoral brain edema

We divided all the examined meningioma cases into 4 subgroups, using Steinhoff classification.

Tryptase expression was observed in: 33 out of 57 meningiomas with I° Steinhoff classification of peritumoral edema, 32 out of 44 meningiomas II° Steinhoff classification, 2 out of 2 meningiomas with III° Steinhoff classification of peritumoral edema. This result was statistically significant (*p* = 0.001) (Table 2).

**Table 2:** Grade of peritumoral brain edema (Steinhoff classification) in correlation with tryptase expression

Tryptase expression	Peritumoral brain edema grade in Steinhoff classification								*p* value
	0 *n* = 51		I *n* = 57		II *n* = 44		III *n* = 2		
(−)	31	20.1 %	24	15.6 %	12	7.8 %	0	0.0 %	*p* = 0.001
(+)	17	11.1 %	24	15.6 %	24	15.6 %	0	0.0 %	
(++)	3	1.9 %	9	5.8 %	8	5.2 %	2	1.3 %	

HIF-1 expression was observed in 40 out of 57 meningiomas with I° Steinhoff classification, in 32 out of 44 with II° Steinhoff classification, and in 2 out of 2 cases with III° Steinhoff classification of peritumoral edema. The result was also statistically significant (*p* = 0.004) (Table 3).

**Table 3:** HIF-1 expression in correlation with the grade of peritumoral brain edema

HIF-1	Peritumoral brain edema grade in Steinhoff classification								*p* value
	0 *n* = 51		I *n* = 57		II *n* = 44		III *n* = 2		
(−)	25	16.3 %	17	11.1 %	12	7.8 %	0	0.0 %	*p* = 0.004
(+)	25	16.3 %	40	26.0 %	31	20.1 %	1	0.6 %	
(++)	1	0.6 %	0	0.0 %	1	0.6 %	1	0.6 %	

## Discussion

Several recent studies have evaluated the mechanisms of peritumoral brain edema, which may complicate diagnosis and treatment of primary brain tumors and metastases [7, 8]. Damage to the BBB through the opening of tight junctions probably plays a significant role in the formation of vasogenic brain edema, which was observed in high grade glial tumors [8]. There are many studies concerning the etiology of the peritumoral brain edema. Our study focused on the influence of hypoxia and presence of mast cells in association with peritumoral brain edema. It is known that hypoxia may increase secretion of cytokines, including interleukins (Il-6), indirectly leading to mast cell degranulation [18]. Also, HIF-1 contributes to inflammatory functions in various components of the innate immune system, like dendritic cells, mast cells or epithelial cells [19].

Meningiomas are common primary brain tumors, but data concerning correlation between grade of meningioma and presence of peritumoral brain edema are inconclusive. Transitional and meningotheliomatous subtypes of meningiomas are often associated with more severe grades of edema [20]. Many authors have attempted to explain the mechanisms of peritumoral brain edema occurrence. Klc et al. [21] suggested that tenascin-extracellular matrix glycoprotein may be responsible for anaplasia, tumor-associated edema and VEGF expression in meningioma cases. Schmid et al. [22] presented VEGF as a crucial factor in angiogenesis in meningiomas, indirectly associated with peritumoral brain edema formation. Recently, it was observed that abnormal expression of occludins, which are normally observed in tight endothelial junctions in the BBB, may be responsible for peritumoral brain edema [23, 24]. Also, the expression of aquaporin-4 is correlated with peritumoral brain edema [25]. Mattei et al. [26] observed that the degree of peritumoral brain edema is associated with the histological grading of meningiomas. However, the results presented by Osawa et al. [27] showed that only unusual types of meningiomas, such as angiomatous, microcystic or secretory, are associated with peritumoral brain edema.

Our results show a statistically significant association between HIF-1 expression, tryptase expression, and the presence of peritumoral brain edema. More than half meningiomas with II° peritumoral brain edema and all cases with III° were positive (statistically significant) for HIF-1 and tryptase expression. Also statistically significant correlation was observed between presence of mast cells (tryptase expression) and HIF-1 expression according to meningioma grade. High grade meningiomas (estimated as G2 or G3) were positive for both proteins, tryptase and HIF-1.

Our results suggest that hypoxia stimulates HIF-1 overexpression as well as mast cell activation, which may lead to blood-brain barrier breakage, resulting in peritumoral brain edema. This theory might be supported by the studies of Kaynar et al. and Jannsen et al. who observed elevated HIF-1 expression in meningiomas, suggesting that other consequences of hypoxia, not only known VEGF activation, are also observed in meningiomas [28–30]. Kan et al. [9] observed a correlation between grade of meningioma and peritumoral brain edema. High grade histology meningioma appeared to be more common in association with edema. Yoo et al. [31] observed in 50 % of all examined meningiomas regions of hypoxia, assessed by expression of carbonic anhydrase 9 (CA9), and this expression was significantly associated with high-grade histology. Also, among low grade meningiomas, CA9 expression tended to be more common in recurrent tumors. Spanberger et al. [32] who examined the expression of HIF-1 in single brain metastases in association with peritumoral brain edema, obtained different results. They concluded that patients with small peritumoral edema have shorter survival times and their tumors are characterized by more brain-invasive growth, lower HIF-1 expression, and less angiogenic activity. Our study presents that high grade meningiomas with significant peritumoral brain edema are very rich in mast cells. Tirakotai et al. [14] described cases of secretory meningiomas with numerous mast cells within the tumors, suggesting that peritumoral brain edema, which was observed in those cases, may be associated with the accumulation and activation of mast cells. Similarly, Bo et al. [13] found numerous inflammatory cells, like lymphocytes, macrophages and mast cells in menigiomas, suggesting that inflammatory cells deserve to be taken into consideration as predictive factors of recurrence and biological aggressiveness of meningiomas. Osawa et al. [27] observed that angiomatous, microcystic, secretory and lymphocyte-rich meningiomas are often associated with higher peritumoral brain edema. Also, Schober et al. [33] found increased numbers of mast cells in microcystic formations in meningiomas that had high peritumoral brain edema.

Despite that low grade meningiomas are mostly benign types of brain tumors, mortality associated with surgery is variable in literature, ranging from 4 to 23 % in the early postoperative period [34]. Presence of peritumoral brain edema is associated with prolonged hospitalization and with postoperative complications, such as intracranial hematoma or intracranial hypertension [34]. Therefore, recognition of the mechanisms responsible for peritumoral brain edema occurrence is very important to improve treatment and later convalescence.

Our study showed that mast cells, which are important in inflammatory processes, as well as HIF-1 may be involved in the formation of peritumoral brain edema, worse prognosis, and more aggressive phenotype of meningioma. Therefore, evaluation of those two markers may be useful in identifying meningiomas with a potentially worse postoperative clinical course.

We confirm that all authors have read and approved the submission of the manuscript. The manuscript has not been published and is not being considered for publication elsewhere, in whole or in part, in any language, except as an abstract. We also declare no conflict of interest, no financial relationships with any industry (through investments, employment, consultancies, stock ownership, honoraria).
